# Cross-task-oriented EEG signal analysis methods: Our opinion

**DOI:** 10.3389/fnins.2023.1153060

**Published:** 2023-03-09

**Authors:** Dong Wen, Zhenhua Pang, Xianglong Wan, Jingjing Li, Xianling Dong, Yanhong Zhou

**Affiliations:** ^1^School of Intelligence Science and Technology, University of Science and Technology Beijing, Beijing, China; ^2^Key Laboratory of Perception and Control of Intelligent Bionic Unmanned Systems, Ministry of Education, Institute of Artificial Intelligence, University of Science and Technology Beijing, Beijing, China; ^3^School of Information Science and Engineering, Yanshan University, Qinhuangdao, China; ^4^Department of Biomedical Engineering, Chengde Medical University, Chengde, China; ^5^School of Mathematics and Information Science and Technology, Hebei Normal University of Science and Technology, Qinhuangdao, China

**Keywords:** EEG signal, cross-task, cross-subject, feature extraction, feature classification

## 1. Introduction

Research on cross-task EEG signals analysis methods has become a fast-growing research hotspot. In recent years, more and more researchers applied the features, which were widely used in EEG signal analysis to cross-task EEG signal analysis studies, including power spectral density (PSD) features (Touryan et al., 2016; Adewale and Panoutsos, 2019), fusion features (Kakkos et al., 2021), etc. The objective aimed to find ways to effectively deal with the differences between tasks. At the same time, some researchers have explored the classifiers which are more friendly to the differences between different tasks by comparing with the traditional feature classification methods, including multi-layer perceptron neural network (MLPNN) (Kamrud et al., 2021), domain adaptive methods (Zhou et al., 2022), sliding-window support vector machine (SVM) (Boring et al., 2020), etc. On the other hand, some new cross-task models based on deep learning models were proposed to narrow the differences between tasks, such as convolutional neural networks (CNNs) (Mota et al., 2021), recurrent neural networks (RNNs) (Gupta et al., 2021), metric-based methods (Jia et al., 2023), combinations of CNNs and RNNs (Zhang et al., 2019; Zhou et al., 2019; Taori et al., 2022), etc. However, there are still many unexplored areas in the field of cross-task EEG signal analysis methods, such as: task segmentation and complexity design (Kamrud et al., 2021), multi-source domain adaptive application (Zhou et al., 2022), multi-scale and multi-directional filter research (Taori et al., 2022), considering both feature extraction and feature classification, and increasing the amount of data. Furthermore, there are also some interconnections between cross-task analysis and relatively common cross-subject studies.

This study will review the literature related to cross-task EEG signal analysis from the perspective of feature extraction and feature classification, and discuss the relationship between cross-task research and cross-subject research for EEG signal analysis, and finally present the point of our original opinion in the purpose of providing useful suggestion for the research field of cross-task EEG signal analysis.

## 2. Cross-task EEG signal analysis based on feature extraction

With the development of EEG signal analysis methods, a series of studies on EEG signal analysis has found that many EEG signal feature extraction methods ignored the interference of different tasks on EEG signal analysis (Xing et al., 2022). Therefore, in order to improve cross-tasks results, more and more researchers are working to find features that perform better on cross-task.

### 2.1. Cross-task EEG signal analysis based on classical features

In EEG signal analysis research, as PSD is one of the most widely used features in EEG signal analysis, some cross-task studies started from PSD features for in-depth exploration. Touryan et al. used ICA to describe feature space, calculated PSD, and identified independent component (IC) sets in spectral properties using sequential forward floating selection (SFFS) (Touryan et al., 2016). The results showed that common components of cross-task EEG signals could be identified through this method. Furthermore, Adewale et al. designed a signal processing and feature extraction framework based on PSD (Adewale and Panoutsos, 2019), and found that PSD could be used as an excellent feature for mental workload estimation. Therefore, PSD features show excellent performance in cross-task EEG signal analysis.

### 2.2. Cross-task EEG signal analysis based on other features

In recent years, it has been found that cross-task EEG signal analysis using PSD features alone does not achieve the best results (Kakkos et al., 2021; Ke et al., 2021; Guan et al., 2022; Xing et al., 2022). Therefore, some studies have begun to use some features combined with PSD or propose new features.

(1) Research based on feature fusion. Kakkos et al. improved the performance of cross-task classification by combining PSD with functional connectivity (FC) features (Kakkos et al., 2021) and demonstrated that the use of brain feature fusion is more effective in cross-task. Ke et al. verified that task-independent auditory event-related potentials (tir-aERPs) have better adaptability than PSD (Ke et al., 2021), and will work on tir-aERPs and PSD feature fusion in their future studies.

(2) Research based on brain network features. Guan et al. proposed a dynamic brain network analysis method based on EEG microstates (Guan et al., 2022) and found that the use of dynamic functional connectivity metrics was more suitable for cross-task.

(3) Research based on fuzzy entropy features. Xing et al. used fuzzy entropy features for cross-task EEG signal analysis (Xing et al., 2022) and found that fuzzy entropy features are more adaptable to cross-tasks than other features.

In summary, the studies of cross-task EEG signals from the perspectives of PSD-based feature fusion and exploration of new features has garnered the attention of numerous researchers, yet further investigation is still required in terms of effective feature fusion and novel cross-task sharing features.

## 3. Cross-task EEG signal analysis based on feature classification

From the perspective of feature classification, although the differences between tasks corresponding to different EEG signals limit the cross-task versatility of existing classification models (Zhou et al., 2022), researchers are still committed to finding or constructing some relatively general cross-task feature classification models (Kamrud et al., 2021; Mota et al., 2021; Taori et al., 2022).

### 3.1. Cross-task EEG signal analysis based on classical classification methods

Classical classification models in the field of EEG signal analysis are emerging, but only a few methods exist in cross-task research field.

(1) Neural network method. Kamrud et al. studied the commonality of three different models in terms of cross-task: MLPNN, temporal convolutional network (TCN), TCN auto encoder (TCN-AE) (Kamrud et al., 2021), and the results showed that the best model for cross-task classification was the MLPNN frequency domain model.

(2) Domain adaptive method. Zhou et al. explored four domain adaptation methods to bridge differences between tasks (Zhou et al., 2022), and the results showed that the transfer joint matching method not only performed best, but always achieved the best performance compared to other methods.

(3) Support vector machine. Boring et al. compared the cross-task classification performance of SVM, linear discriminant analysis (LDA), and k-nearest neighbors (KNN) under sliding window (Boring et al., 2020), and the results showed that the performance of SVM was significantly better than that of other models.

The above studies analyzed the performance of some classical methods in the application of cross-task EEG signal analysis, and there may be some classical methods with better performance in the future that can be used for cross-task EEG signal analysis.

### 3.2. Cross-task EEG signal analysis based on other classification methods

In addition to the above classical methods, the following methods have also achieved excellent performance in cross-task EEG signal analysis.

(1) CNN. Mota et al. proposed a cross-task classification method based on CNN and compressed excitation blocks (Mota et al., 2021), and the results showed that compressed excitation blocks could be used to explore the dependence on EEG signal pathways.

(2) RNN. Gupta et al. proposed a deep RNN model (Gupta et al., 2021), which showed that the model could learn forward and reverse temporal dynamics and had long-term memory ability.

(3) Multi-classifier combination. Zhou et al. proposed a cross-task method for classification using raw data (Zhou et al., 2019), and the results showed that the method had good adaptability. In the same year, Wang et al. proposed a cascade structure (R3DCNN) of a deep recurrent and three-dimensional convolutional neural network (3DCNN) (Zhang et al., 2019), and the results showed that 3DCNN could be used to learn the spatial and spectral features of EEG signals, and the use of RNN layers that can obtain temporal representations improved the performance. Taori et al. proposed a structural model built on RNN and attention mechanisms (Taori et al., 2022), and the results showed that the model could extract effective cross-task features from the space-time domain.

(4) Metric-based method. Jia et al. proposed a metric-based Spatial Filtering Transformer (MSFT) model, which used the angle margin loss function (Jia et al., 2023), and the results showed that the method had good application prospects in the field of cross-task EEG signal analysis.

The above methods showed excellent performance in cross-task research, and future research could tend to build new cross-task classification models.

## 4. Study on cross-task and cross-subject relationship for EEG signal analysis

The significant variability of EEG signals between individuals reduces the generalization ability of EEG analysis algorithms (Xu et al., 2021). Since 2005 or even earlier, cross-subject EEG signals analysis has flourished, a variety of cross-subject methods has been designed, Tangermann et al. proved that recursive channel elimination (RCE) can be used for cross-subject combinatorial data analysis (Tangermann et al., 2005), and Dyson et al. conducted cross-subject studies by sequential forward-floating search algorithms (Dyson et al., 2010). On the other hand, cross-task research has only been in its infancy in recent years, and the current cross-task approach has a mutually reinforcing relationship with the existing cross-scenario approach.

(1) Cross-subject methods are innovatively applied to cross-task research. In 2012, Khalighi et al. proposed a cross-subject method for unsupervised domain adaptation (Khalighi et al., 2012). In 2021, Zhao et al. proposed an aligned multi-source domain adaptation method for cross-subject (Zhao et al., 2021). Zhou et al. proposed a cross-task domain adaptive method based on the above (Zhou et al., 2022). In 2016, Hajinoroozi et al. proposed a cross-subject method for channel convolutional neural networks (Hajinoroozi et al., 2016), and 5 years later, Mota et al. proposed a CNN-based cross-task method (Mota et al., 2021). Similarly, in 2019, Hang et al. achieved feature fusion of cross-subject EEG signals (Hang et al., 2019), and 2 years later, Kakkos et al. also explored feature fusion of cross-task EEG signals (Kakkos et al., 2021).

(2) Cross-task methods are innovatively applied to cross-subject research. In 2016, Touryan et al. studied the PSD features of cross-task EEG signals (Touryan et al., 2016), and 2 years later, Booth et al. carried out in-depth research on the PSD features of cross-subject EEG signals (Booth et al., 2018).

In summary, there is a certain correlation between cross-task and cross-individual research methods, and combining cross-task and cross-subject research will make EEG analysis methods more versatile.

## 5. Discussion

In this study, the cross-task EEG signal analysis method was analyzed from three aspects: feature extraction, feature classification, and the relationship between cross-task and cross-subject methods. While these studies have yielded promising results, more exploration is needed before confident conclusions can be drawn. Therefore, this paper raises our own opinion on the future research of cross-task EEG signal analysis.

(1) Increasing the sample size. In the future, more EEG data can be collected or data augmentation techniques can be used to increase the sample size in order to improve the generalization performance of cross-task methods.

(2) Research from the perspectives of both feature extraction and feature classification. At present, most cross-task researches were carried out independently from the perspective of feature extraction or feature classification, and it is a valuable practice to find common ground from these two perspectives simultaneously in the future, such as multi-source domain adaptation (Zhou et al., 2022) and multi-scale and multi-directional filter (Taori et al., 2022) used for the study of single cross-task EEG signals.

(3) Task subdivision. In the future, it is necessary to subdivide different tasks to improve the practicability of cross-task research, such as the corresponding EEG signal datasets can be analyzed on tasks with the same cognitive domain but different cognitive training content, or tasks with different cognitive domains and different cognitive training content.

(4) The research of cross-task regression models can be explored in depth. The study results of Ke et al. suggested that regression models rather than classifiers should be used for obtaining optimal results in some cross-task studies (Ke et al., 2014). In the future, we can try to explore cross-task research on other types of regression models.

In conclusion, this paper introduced some research trends in the future. If cross-task research can continue to advance in these areas, it will take this type of research to a higher level.

## Author contributions

DW, ZP, and YZ contributed to conception and design of the study. ZP searched the database. DW, ZP, and JL performed the analysis of literatures. DW and ZP wrote the first draft of the manuscript. XW and JL wrote sections of the manuscript. YZ and XD revised this paper. All authors contributed to manuscript revision, read, and approved the submitted version.
